# *Solanum trilobatum* L. Ameliorate Thioacetamide-Induced Oxidative Stress and Hepatic Damage in Albino Rats

**DOI:** 10.3390/antiox6030068

**Published:** 2017-08-22

**Authors:** Kumar Ganesan, Kumeshini Sukalingam, Baojun Xu

**Affiliations:** Food Science and Technology Program, Beijing Normal University–Hong Kong Baptist University United International College, Zhuhai 519085, China; kumarganesan@uic.edu.hk (K.G.); meshni_anat@yahoo.com.my (K.S.)

**Keywords:** *Solanum trilobatum*, antioxidant enzymes, thioacetamide, liver toxicity

## Abstract

*Solanum trilobatum* L. (Solanaceae) has been well known as nightshade, commonly used by diverse populations to heal several disorders. Earlier studies in *Solanum trilobatum* were focused on different pharmacological activities and a few were concerned with antioxidant and hepatoprotective effects. Thus, the current study was focused to evaluate the antioxidant potential and hepatoprotective effects of *S. trilobatum* L. on thioacetamide (TAA) intoxication in Wistar albino rats. The rats were kept into four groups and six animals each. Group A was normal control. Group B was the TAA treated control. Groups C and D were pretreated with the aqueous extract from the leaves of *S. trilobatum* (100 mg, 200 mg/kg bw p.o.) once daily for 10 consecutive days administration followed by a single dose infusion of TAA (100 mg/kg s.c.). After 10 days, blood and livers were collected. The biochemical assay was carried out in the GSH (reduced glutathione), TBARS(thiobarbituric acid reactive substances), Na^+^-K^+^-ATPase, and antioxidant enzymes viz., SOD (superoxide dismutase), CAT (catalase), GPx (glutathione peroxidase), GST (glutathione-S-transferase), and GR (glutathione reductase) were analyzed in samples of blood and liver. Treatment with *S. trilobatum* reduced blood and liver TBARS, and Na^+^ K^+^ ATPase activity in TAA (thioacetamide)-induced hepatotoxicity rats. Furthermore, the above antioxidant enzymes were increased in the pretreatment of *S. trilobatum* in TAA intoxicated rats. Finally, we concluded that *S. Trilobatum* displayed potent antioxidant properties and alleviate oxidative stress induced hepatotoxic effects and possible engross mechanisms related to free radical scavenging properties.

## 1. Introduction

Currently, oxidative stress associated free-radical biology have become niche research attention. Free radicals play a crucial function in the progress of tissue damage and pathological events in living beings [1]. In a cell, lipid peroxidation is restricted by several cellular defense mechanisms consisted of enzymatic and non-enzymatic scavenging systems [2]. Recent studies have also specified that there is a contrary connection between the intake of food rich in antioxidants and the occurrence of human illness [3]. Thus the investigations of new synthetic or natural antioxidants are urgently important.

The properties of medicinal plants have been investigated in the recent scientific progress due to its effective antioxidant potentials and ability in healing various ailments. Normally, antioxidants inhibit oxidative stress related tissue damage caused by free radicals; either obstruct oxidation mechanisms or scavenge the oxygen [4,5]. Potentially reactive oxygen derivatives are known to be recognized as reactive oxygen species (ROS), which is consistently produced by various biochemical mechanisms in the human cells. However, these generated ROS are detoxified by the antioxidants, either present in the body or in antioxidant rich food. 

ROS generally caused by O_2_•^−^, HO• and H_2_O_2_ and led to the alteration of the enzymatic antioxidant defense systems in in vitro and in vivo models [6]. SOD, CAT, GSH, GPx, GR, and GST are the primary antioxidant enzymes serve as redox biomarkers as they are the first-line indicators of the antioxidant state through oxidation/reduction processes [7,8]. As the most abundant intracellular antioxidant, GSH is involved in the protection of cells against oxidative damage and in various detoxification mechanisms [9]. GSH primarily acts as a substrate and co-substrate in many essential enzymatic reactions involving GPx, GR, and GST, and a decrease in the GSH level usually impairs cells’ response to oxidants [10]. Conversely, excess ROS generation and/or insufficient antioxidant defense can simply influence oxidative injury to diverse biomolecules including proteins, lipids, and DNA [11]. These oxidative damages cause many chronic diseases in human including diabetes, cancer, arthritis, cardiovascular diseases, neurodegenerative diseases, and aging [12]. Numerous compounds have been used as hepatotoxic models, which facilitate investigation on a variety of pathological features. A single administration of thioacetamide (TAA) at a dose of 100 mg/kg causes hepatic centrilobular necrosis, cirrhosis, and fibrosis in the rodents [13,14,15]. Further, the study showed that prolonged administration of TAA along with diet causes liver cancer [16].

*S. trilobatum* Linn (Solanaceae) is a known shrub, familiar as ‘Tuduvelai’ and is distributed in various Asian countries, including India, Sri Lanka, Indonesia, Singapore, and Malaysia [17]. In Ayurveda and Siddha medicinal systems, the roots and leaves are prescribed to heal various respiratory tract problems, including acute and chronic bronchitis, asthma, sinusitis, tonsillitis, common cold, cough, and pulmonary infections [18,19,20,21,22]. The leaves are mainly used in treating dyspepsia, spermatorrhoea, tuberculosis, ear problems, and bacterial infections [23]. *S. trilobatum* has been extensively studied for various pharmacological activities including antibacterial, antifungal, anticancer [24,25,26,27,28,29,30,31,32,33], antioxidant [34], antidiabetic [35], hepatoprotective [27], antinociceptive [36], anti-ulcerogenic [37], anti-inflammatory [38,39,40,41,42,43], and mosquitocidal activity [41,42]. Studies showed that the leaves contain various metabolites, including sugar, fat, fiber, calcium, phosphorus, ferrous, and other minerals [38]. Phytochemical screening of the dried leaves found alkaloids, triterpenoids, phenolics, tannins, flavonoids, anthoquinones, phytosterols, saponins, cardiac glycosides, soladunalinidine, tomatidine, solanine, sobatum, solasodine, diosgenin, and β-solamarine [37,44]. Based on the literature, few studies were concerned with the investigation on antioxidant and hepatoprotective effects. Thus, the current study aimed to evaluate the antioxidant potential and hepatoprotective effects of *S. trilobatum* L. on TAA intoxication in Wistar albino rats.

## 2. Materials and Methods

### 2.1. Animals

Male Wister albino rats weighing about 180–200 g were used in the present study. Animals were procured from the animal house of Management and Science University, Malaysia and were kept in wire-floored cages under normal laboratory settings in 12 h light/dark cycles at 25–28 °C and 60–80% relative humidity. Animals were nurtured at robust health by the supply of a normal pellet diet and water ad libitum. Animal studies were carried out in accordance with the National Institute of Health Guide [45]. The study was initially approved by the ethics committee (The committee for the purpose of control and supervision of experiments on animals-CPCSEA) and ethical norms were strictly followed during all experimental procedures (Reg No. 12/2011/CPCSEA, proposal No. 77).

### 2.2. Plant Material

The fresh leaves of *S. trilobatum* L. were collected during January–February 2014 from Sri Muda, Shah Alam, Selangor, Malaysia. The plant was authenticated by Dr. Sujit, Taxonomist in the Department of Pharmacognosy and compared with reference specimens preserved in the Herbarium and Center for Molecular Systematics in Management and Science University, Malaysia. Voucher specimens are maintained in the institution.

### 2.3. Preparation of Plant Extract

Fresh powdered leaves (500 g) were subjected to 2 L of distilled water and extraction was kept in a cold room with constant stirring overnight. The extraction was filtered using cheesecloth, and Whatmann filter paper followed by centrifugation (1200× *g* for 10 min). The supernatant was evaporated under reduced pressure using a vacuum rotary evaporator and residues were kept under refrigeration until used (yield: 210 w/w).

### 2.4. Chemicals

Thioacetamide (TAA), thiobarbituric acid (TBA), 1,1-diphenyl-2-picrylhydrazyl (DPPH), 5,5-dithiobis-2-nitrobenzoic acid (DTNB), nicotinamide adenine dinucleotide hydrogen (NADH), phenazine methosulphate, trichloroacetic acid (TCA), 1-chloro-2, 4-dinitrobenzene (CDNB), nicotinamide adenine dinucleotide hydrogen phosphate (NADPH) were obtained from Sigma-Aldrich Co., St. Louis, MO, USA, and Merck (Darmstadt, Germany). All chemicals and reagents used in the experiments were of analytical grade (AR).

### 2.5. Experimental Design

Animals were kept into four groups and six rats each. Group A was normal control. Group B was TAA treated control. Group C and D were pretreated with the aqueous extract from the leaves of *S. trilobatum* (100 mg, 200 mg/kg bw p.o.) once daily for 10 consecutive days administration followed by a single dose infusion of TAA (100 mg/kg s.c.) as a 2% *W*/*V* solution in distilled water. The control group received the vehicle as distilled water. The method of acute hepatotoxicity induction was followed according to the method of Kumar et al. [46]. After 24 h of toxin administration, all rats were sacrificed by cervical dislocation; blood was collected and used for the measurement of antioxidant enzymes. Livers were dissected immediately and homogenized. The homogenates were then centrifuged at 3200× *g* for 20 min at 4 °C and supernatant obtained was used for the assay of various enzymes.

### 2.6. Assay of Antioxidant Enzymes

#### 2.6.1. Reduced Glutathione (GSH) Assay

GSH was estimated using DTNB by the method of Sedlak and Lindsay [47]. 1 mL sample of 10% homogenate was precipitated with 1 mL of (4%) sulfosalicylic acid. The samples were maintained at 4 °C for 1 h and then centrifuged at 1200× *g* for 20 min at 4 °C. Added 0.1 mL filtered aliquot, 0.2 mL of 100 mM DTNB, 2.7 mL phosphate buffer (0.1 M, pH 7.4) in the cuvette. The yellow color of the mixture was developed, read immediately at 412 nm on the spectrophotometer and expressed as μM GSH/g tissue. 

#### 2.6.2. Superoxide Dismutase (SOD) Assay

SOD activity was estimated by the method of Kakkar et al. [48]. Reaction mixture of this method contained 0.1 mL of phenazine methosulphate (186 μM), 1.2 mL of sodium pyrophosphate buffer (0.05 mM, pH 7.0), 0.3 mL of supernatant after centrifugation (1500× *g*, 10 min followed by 10,000× *g*, 15 min) of 10% homogenate was added to the reaction mixture. The enzyme reaction was initiated by adding 0.2 mL of NADH (780 μM) and stopped after 1 min by adding 1 mL of glacial acetic acid. The changes in the absorbance were measured at 560 nm using the spectrophotometer. Results are expressed in units/mg protein.

#### 2.6.3. Catalase Activity (CAT)

CAT activity was measured by following decomposition of H_2_O_2_ according to the method of Aebi and Vergmeyer [49]. The reaction solution contained 0.1 mL enzyme extract, 2.5 mL of 50 mM phosphate buffer (pH 5.0), and 0.4 mL of 6 mM H_2_O_2_. After one min, the absorbance of the reaction solution was read at 240 nm. One unit of CAT activity was defined as an absorbance change of as 0.01 units/min.

#### 2.6.4. Glutathione Peroxidase (GPx) Assay

GPx activity was assayed by the method of Rotruck et al. [50] using H_2_O_2_ as substrate. The reaction mixture consisted of 1.49 mL phosphate buffer (0.1 M, pH 7.4), 0.1 mL sodium azide (1 mM), 0.05 mL glutathione reductase (1 IU/mL), 0.05 mL GSH (1 mM), 0.1 mL EDTA (1 mM), 0.1 mL NADPH (0.2 mM), 0.01 mL H_2_O_2_ (0.25 mM), and 0.1 mL 10% homogenate in a total volume of 2 mL. The discoloration of NADPH at 340 nm was recorded at 25 °C. Enzyme activity was calculated as nM NADPH oxidized/min/mg protein using a molar extinction coefficient of 6.22 × 10^3^/M cm.

#### 2.6.5. Glutathione-S-Transferase (GST) Assay

GST activity was measured using CDNB as substrate according to Habig et al. [51]. The reaction mixture consisted of 1.475 mL phosphate buffer (0.1 M, pH 6.5), 0.025 mL CDNB (1 mM), 0.2 mL reduced GSH (1 mM), and 0.3 mL of 10% homogenate in a total volume of 2.0 mL. The absorbance was measured at 340 nm using a spectrophotometer and GST activity was determined as nM CDNB conjugate formed/min/mg protein using a molar extinction coefficient of 9.6 × 10^3^/M cm.

#### 2.6.6. Glutathione Reductase (GSR) Assay

GSR activity was determined by the method of James et al. [52]. The reaction solution composed of 1.65 mL phosphate buffer: (0.1 M, pH 7.6), 0.1 mL EDTA (0.5 mM), 0.1 mL NADPH (0.1 mM), 0.05 mL oxidized glutathione (1 mM), and 0.1 mL 10% homogenate in a total volume of 2 mL. Enzyme activity was estimated at 25 °C by measuring the disappearance of NADPH at 340 nm and was calculated as nM NADPH oxidized/min/mg protein using a molar extinction coefficient of 6.22 × 10^3^/M cm.

#### 2.6.7. Estimation of Lipid Peroxidation (TBARS) Assay

The TBARS assay for lipid peroxidation was carried out by following the method of Ohkawa et al. [53]. The reaction mixture in a total volume of 1 mL contained 0.58 mL phosphate buffer (0.1 M, pH 7.4), 0.2 mL homogeneous samples, 0.2 mL ascorbic acid (100 mM), and 0.02 mL ferric chloride (100 mM). The composite was kept at 37 °C for 1 h in a water bath. The reaction was stopped by the addition of 1.0 mL 10% TCA. After addition of 1.0 mL 0.67% TBA, all the tubes were boiled in a water bath for 20 min and then shifted to an ice-bath before centrifuging at 2500× *g* for 10 min. The quantity of TBARS was analyzed by determining the optical density of the supernatant at 535 nm using a spectrophotometer. The results were expressed as nM TBARS/min/mg tissue at 37 °C using a molar extinction coefficient of 1.56 × 10^5^/M cm. The concentration of TBARS was expressed as nmol of malondialdehyde per mg of protein using 1,1,3,3-tetraethoxypropane as the standard.

#### 2.6.8. Measurement of Na^+^ K^+^ ATPase Activity 

Na^+^ K^+^ ATPase activity was measured by Bonting method [54]. This method is based on the measurement of orthophosphate released from ATP during incubation of membranes with a medium containing: 1 mM ATP, 10 mM MgCl_2_, 100 mM Tris-HCl buffer (pH 7.4), and 0.1 mM ouabain, which is added to block the Na^+^ K^+^ ATPase. Samples were incubated for 30 min at 37 °C and of 0 °C. After the incubation, 0.6 M TCA was added. The concentration of the orthophosphate was determined in the supernatant by the Veldhoven and Mannaers’ method.

### 2.7. Statistical Analysis

All data obtained in the study were expressed as Mean ± S.D., using the Statistical Package for Social Sciences (SPSS) software made for Windows Version 20.0 and statistically assessed by one-way analysis of variance (ANOVA). The difference between test animals and controls were evaluated by Student’s *t*-test [55].

## 3. Results

The level of glutathione (GSH) in the blood and in liver homogenate, liver Na^+^ K^+^ ATPase and liver TBARS in TAA intoxicated rats were given in Table 1. Animals treated with TAA elevated blood and liver GSH, and Na^+^K^+^ATPase level. Pretreatment of rats with 100 mg, 200 mg/kg bw of aqueous leaf extracts of *S. trilobatum* showed a significant reclamation in the reduction of blood (75%) and liver GSH (70%), liver Na^+^ K^+^ ATPase level (75.7%). While TBARS of TAA treated animals were significantly higher than the control animals. Administration of aqueous extract of leaves of *S. trilobatum* markedly decreased the level of TBARS (85.6%). The protection was maximal with the treatment at a higher dose of the extract.

The effect of aqueous extract of leaves of *S. trilobatum* on GSR, GST, GP_X_, SOD, and CAT in TAA intoxicated rats were shown in Table 2. Levels of these antioxidant enzymes were decreased significantly (*p* < 0.001) in TAA-induced rats when compared with those of control animals. Treatment of rats with aqueous extract from leaves of *S. trilobatum* (100 mg, 200 mg/kg bw p.o.) showed an elevated level of those antioxidant enzymes such as GSR (69.6%), GST (67.7%), GPx (87.5%), SOD (75.7%), and CAT (88.7%) in TAA intoxicated rats. The hepatoprotection was higher with the treatment at a higher dose of the plant extracts.

## 4. Discussion

Generally, glutathione plays a noteworthy cellular antioxidant defense mechanism that scavenges singlet oxygen, superoxide, and hydroxyl radicals [1,2]. The degradation of the GSH-dependent antioxidant defense mechanism enhances the intracellular flux of oxygen radicals [3] which creates cellular oxidative stress and promoting apoptosis [56]. The protective functions of GSH on cellular lipid peroxidation have been well documented [5]. An increase hepatic lipid peroxidation was evident by lifting MDA in liver homogenate and its reduction to nearly normal levels by *S. trilobatum* administration.

Thioacetamide was the well-known hepatotoxin and causes hepatocellular carcinoma in rodents [46]. The studies further suggested that the acute or chronic exposure of TAA caused liver cirrhosis, encephalopathy, and fibrosis in rats [13,14,15,16]. In the present findings, TAA produced depletion of blood and liver GSH and elevation of hepatic TBARS. Pretreatment of rats with *S. trilobatum* (100, 200 mg/kg bw) significantly abridged the TBARS levels and increased the concentration of GSH. These findings demonstrated that a high concentration of GSH in blood and liver could provide a strong tissue defense system against oxidative stress, and thus contribute to attenuating the hepatotoxicity effect of TAA. In addition, detoxification of TAA in the liver could be triggered by GST-catalyzed conjugation with GSH [57], the elevated levels of GST in the liver persuaded by the administration of crude extracts of *S. trilobatum*, as a result, diminish the acute hepatotoxicity effect of TAA. The GSR is a member of cytosolic enzymes, mainly involved in the detoxification of xenobiotic compounds by their GSH conjugation [58]. It has been reported that TAA produces an increased blood GSR activity, which occurs at a maximum of 24 h after administration. Pre-treatment of *S. trilabatum* significantly reduced GSR in TAA administered rats.

The enzyme activities of Na^+^ K^+^ ATPase, SOD, and CAT were significantly decreased (*p* < 0.001), the experiment was positively correlated with previous studies associated with hepatotoxin-treated animals [57,59]. The administration of plant extract showed the reversal of these enzyme activities and could be helpful to reduce the hepatotoxin-induced liver damage. These findings suggested that pre-treatment of leaf extract of *S. trilabatum* elevated the activity of SOD and CAT, and thereby inhibited the generation of lipid peroxides and reduced hepatic damage. In addition, these elevated levels of enzymes in *S. trilobatum* treated animals has prominent free radical scavenging activity, which may exert a beneficial effect against pathological alterations, and protect the membrane damage in liver caused by ROS. Antioxidants may be molecules/substances that can neutralize free radicals by accepting or donating electron(s) to eliminate the unpaired condition of the radical [7,8,59]. *S. trilobatum* possesses enormous bioactive substances, acts as antioxidant molecules, which may directly react with the reactive radicals and destroy or neutralize them [60]. Hence, the present findings strongly suggest that the hepatoprotective action of the plant extract might be due to its antioxidant potential. However, the potential of antioxidant agents and active principles can only be realized after further comprehensive pharmacological and molecular mechanisms. 

## 5. Conclusions

In conclusion, the current finding highlights that *S. trilobatum* is known to be possess potent antioxidants and ameliorate the effect of TAA-induced oxidative stress and liver toxicity in rats appears to be associated with the inhibition of lipid peroxides and elevation the antioxidant enzymes in the blood and liver.

## Figures and Tables

**Table 1:** Effect of aqueous extract of *Solanum trilobatum* on GSH (blood & liver), liver Na^+^ K^+^ ATPase, and liver TBARS in rats subjected to TAA toxicity.

Treatment	Blood GSH (mg%)	Liver GSH (μmol/g of Liver)	Liver Na^+^K^+^ATPase (U/g prOtein)	TBARS (Nmol MDA/g of Wet Tissue/h)
Control	2.82 ± 0.06	11.2 ± 0.56	13.6 ± 1.23	359.42 ± 18.45
TAA	0.98 ± 0.02 ^a^	7.89 ± 0.56 ^b^	8.56 ± 1.03 ^b^	498.5 ± 15.69 ^a^
*S. trilobatum* (100 mg/kg bw)	1.96 ± 0.04 ^c^ (53.3)	8.95 ± 1.06 ^d^ (32)	10.57 ± 1.13 ^c^ (40)	409.6 ± 19.46 ^c^ (64)
*S. trilobatum* (200 mg/kg bw)	2.36 ± 0.56 ^c^ (75)	10.2 ± 1.23 ^c^ (70)	12.65 ± 1.64 ^c^ (75.7)	379.4 ± 19.49 ^c^ (85.6)

Values are mean ± S.D.; *n* = 6 animals per group. Values in the parental protection in individual biochemical parameters from their elevated values caused by the hepatoprotection. The percentage of protection is calculated as 100 × (values of TAA control values of sample)/(values of TAA control − values of control). ^a^ *p* < 0.001 when compared with Group A; ^b^ *p* < 0.01 when compared with Group A; ^c^ *p* < 0.001 when compared with Group B; ^d^ *p* < 0.01 when compared with Group B; MDA: melondialdehyde.

**Table 2:** Effect of aqueous extract of *Solanum trilobatum*. L on GSR, GP_X_, GST, SOD, and CAT in rats subjected to TAA toxicity.

Treatment	GSR (µmol NADPH/min/g/of Wet Liver)	GPx (U/mg Protein)	GST (U/g of Wet Weight)	SOD (U/mg Protein)	CAT (H_2_O_2_ Decomposed/min/mg Protein)
Control	189.5 ± 10.8	13.46 ± 1.78	156.4 ± 9.43	10.56 ± 1.89	90.25 ± 8.2
TAA	122.6 ± 12.8 ^a^	8.97 ± 1.02 ^b^	88.9 ± 5.6 ^b^	5.57 ± 0.96 ^a^	55.96 ± 8.7 ^a^
*S. trilobatum* (100 mg/kg bw)	155.8 ± 15.6 ^d^ (50)	10.9 ± 1.46 ^d^ (43)	116.9 ± 9.87 ^d^ (41.4)	8.9 ± 1.72 ^d^ (66.7)	72.8 ± 9.8 ^c^ (49.1)
*S. trilobatum* (200 mg/kg bw)	169.2 ± 10.3 ^c^ (69.6)	12.9 ± 1.08 ^c^ (87.5)	134.6 ± 10.6 ^d^ (67.7)	9.35 ± 0.27 ^c^ (75.7)	86.4 ± 9.89 ^c^ (88.7)

Values are mean ± S.D.; *n* = 6 animals per group. Values in the parental protection in individual biochemical parameters from their elevated values caused by the hepatoprotection. The percentage of protection is calculated as 100 × (values of TAA control values of sample)/(values of TAA control − values of control). ^a^ *p* < 0.001 when compared with Group A; ^b^ *p* < 0.01 when compared with Group A; ^c^ *p* < 0.001 when compared with Group B; ^d^ *p* < 0.01 when compared with Group B.

## Abbreviation

ATP	adenosine triphosphate
b.w	body weight
CAT	catalase
CDNB	1 chloro 2, 4 dinitrobenzene
cm	centimeter
DNA	deoxy ribonucleic acid
DPPH	1,1-diphenyl-2-picrylhydrazyl
DTNB	5,5-dithiobis-2-nitrobenzoic acid
EDTA	ethylene diamine tetra acetic acid
GPx	glutathione peroxidase
GR	glutathione reductase
GSH	reduced glutathione
GST	glutathione-S-transferase
HO•	hydroxyl radicals
H_2_O_2_	hydrogen peroxide
Kg	kilogram
MDA	melondialdehyde
mg	milligram
MgCl_2_	magnesium chloride
mM	millimolar
Na^+^-K^+^-ATPase	sodium-potassium adenosine triphosphatase
NADH	nicotinamide adenine dinucleotide hydrogen
NADPH	nicotinamide adenine dinucleotide hydrogen phosphate; nm-nanometer
O_2_•	oxygen radicals
°C	degree Celsius
p.o.	per oral
ROS	reactive oxygen species
s.c.	subcutaneous
S.D.	standard deviation
SOD	superoxide dismutase
TAA	thioacetamide
TBA-	thiobarbituric acid
TBARS	thiobarbituric acid reactive substances
TCA	trichloroacetic acid
μM	micro molar
